# Efficacy and safety of acupuncture for chemotherapy-induced nausea and vomiting: a systematic review and meta-analysis of randomized controlled trial

**DOI:** 10.3389/fneur.2026.1774507

**Published:** 2026-07-03

**Authors:** Jiongli Chen, Fangyue Xu, Lingyu Zhang, Shengyang Hu, Yaxuan Xu, Qiongbo Hua, Haitao Chen, Rongrong Li

**Affiliations:** 1 The Third Clinical Medical College of Zhejiang Chinese Medical University, Hangzhou, China; 2 The Second Clinical Medical College of Zhejiang Chinese Medical University, Hangzhou, China; 3 Department of Oncology, The Second Affiliated Hospital of Zhejiang Chinese Medical University, Hangzhou, China; 4 The Third Affiliated Hospital of Zhejiang Chinese Medical University, Hangzhou, China

**Keywords:** acupuncture, chemotherapy-induced nausea and vomiting, meta-analysis, nausea, systematic review, vomiting

## Abstract

**Objective:**

Chemotherapy-induced nausea and vomiting (CINV) represents a common and debilitating side effects in cancer patients, often compromising treatment adherence and quality of life. Current management remains suboptimal, necessitating the exploration of complementary therapies. Acupuncture, as a widely used adjunctive therapy, has been attempted for the treatment of CINV. This study aims to systematically evaluate the effect and safety of acupuncture in managing CINV.

**Methods:**

We comprehensively searched seven electronic databases (EMBASE, Cochrane Library, Web of Science, PubMed, China National Knowledge Infrastructure, VIP Chinese Science and Technology Periodicals Databas, and Wanfang) and two clinical trial registries (ClinicalTrials.gov, Chinese Clinical Trial Registry) for randomized controlled trials (RCTs) comparing acupuncture with sham acupuncture or conventional treatment for CINV. The primary outcomes included complete response rate (no vomiting episodes plus no or mild nausea), the frequency of vomiting episodes, and validated scale scores. Studies quality were assessed by using the Cochrane Risk of Bias tools.The registration number of this study is CRD420251102130.

**Results:**

49 RCTs involving 4,133 participants were included in this study. The results demonstrated that compared with the control group, acupuncture significantly reduced the incidence of vomiting (RR = 0.583, 95% CI: 0.523–0.650), vomiting severity (MD = −0.839, 95% CI: −1.256 to −0.422) and vomiting episodes (MD = −3.704, 95% CI: −6.256 to −1.152). Additionally, acupuncture lowered the incidence of nausea (RR = 0.532, 95% CI: 0.432–0.655), and nausea severity (MD = −0.895, 95% CI: −1.273 to −0.516). The most utilized acupoints were Zusanli (ST36, *n* = 42), Neiguan (PC6, *n* = 38), and Zhongwan (CV12, *n* = 23).

**Conclusion:**

This study confirms that acupuncture is effective as an adjunctive therapy for CINV. While future large-scale, rigorously designed RCTs are warranted to further validate these findings, the current evidence provides a robust rationale for its integration into clinical practice.

**Systematic review registration:**

The registration number of this study is CRD420251102130.

## 1 Introduction

Cancer remains one of the most significant global health challenges, with the persistently rising incidence and mortality rates creating substantial socioeconomic burdens on healthcare systems and society (1, 2). Chemotherapy serves as cornerstone therapy in multimodal cancer treatment and has significantly improved patient survival outcomes. However, its clinical utility is often limited by substantial toxicities, particularly Chemotherapy-induced nausea and vomiting (CINV).

Approximately 40–80% of patients experience CINV (3, 4), making it one of the most debilitating and treatment-limiting adverse effects. CINV is classified into five subtypes based on the timing: acute, delayed, anticipatory, breakthrough, and refractory (5). The underlying pathophysiology involves neurotransmitters such as serotonin (5-HT) and substance P, which stimulate the chemoreceptor trigger zone (CTZ) and vagal afferent pathways, ultimately activating the emetic center to initiate the vomiting reflex (6, 7). CINV leads to serious clinical sequelae, including anorexia, malnutrition, electrolyte disturbances, and dehydration (8). Moreover, it contributes to chemotherapy non-adherence, unplanned hospital admissions, and significant socioeconomic burdens due to lost productivity and increased healthcare expenditures (9). Consequently, optimal CINV management has evolved from simple symptom relief to an essential component of comprehensive supportive care throughout chemotherapy.

Current treatment of CINV is mainly based on a risk-stratified, multidrug prophylactic egimen. Among these, the first-line therapy include oral antiemetics, such as 5-HT3 receptor antagonists, NK-1 receptor antagonists, corticosteroids, and the atypical antipsychotic olanzapine (7, 10). Despite these interventions, approximately 30% of patients still experience breakthrough CINV (4, 7), highlighting the limitations of existing pharmacotherapy. Beyond suboptimal efficacy, prolonged or combined use of antiemetics may cause adverse effects, such as weight gain. Additionally, high-cost targeted agents, such as NK-1 receptor antagonists, impose a significant economic burden on patients and healthcare systems (11, 12). Given these challenges, integrating complementary adjuvant therapies with conventional antiemetics warrants further exploration.

Acupuncture, a widely used in Traditional Chinese Medicine, has garnered increasing interest as an adjunctive therapy for various conditions due to its favourable safety profile, cost-effectiveness, and high patient acceptability (13). While the National Comprehensive Cancer Network (NCCN) guidelines include acupuncture as a recommended option for CINV management, its clinical efficacy remains a subject of ongoing debate (14). Early investigations proposed that electroacupuncture (EA) stimulation at specific acupoints could ameliorate CINV symptoms (15), a finding subsequently corroborated by several studies (16). Recent high-quality randomized controlled trials (RCTs) have further strengthened this evidence, demonstrating superior CINV control when EA is combined with standard antiemetic regimens (17). However, contradictory findings other studies reporting no significant benefit over conventional therapy alone have introduced uncertainty (18). These discrepancies may be attributable to methodological limitations, particularly concerning adequate blinding procedures, which merit careful scrutiny.

This study aims to systematically evaluate the efficacy and safety of acupuncture in treating CINV, with the intention of providing a reference for future clinical practice.

## 2 Method

This study protocol was registered with the International Prospective Register of Systematic Reviews (PROSPERO) under the registration number CRD420251102130.^1^ The study process was conducted in strict accordance with the Preferred Reporting Items for Systematic Reviews and Meta-Analyses (PRISMA) guidelines (19).

### 2.1 Retrieval strategy

**Figure 1:**
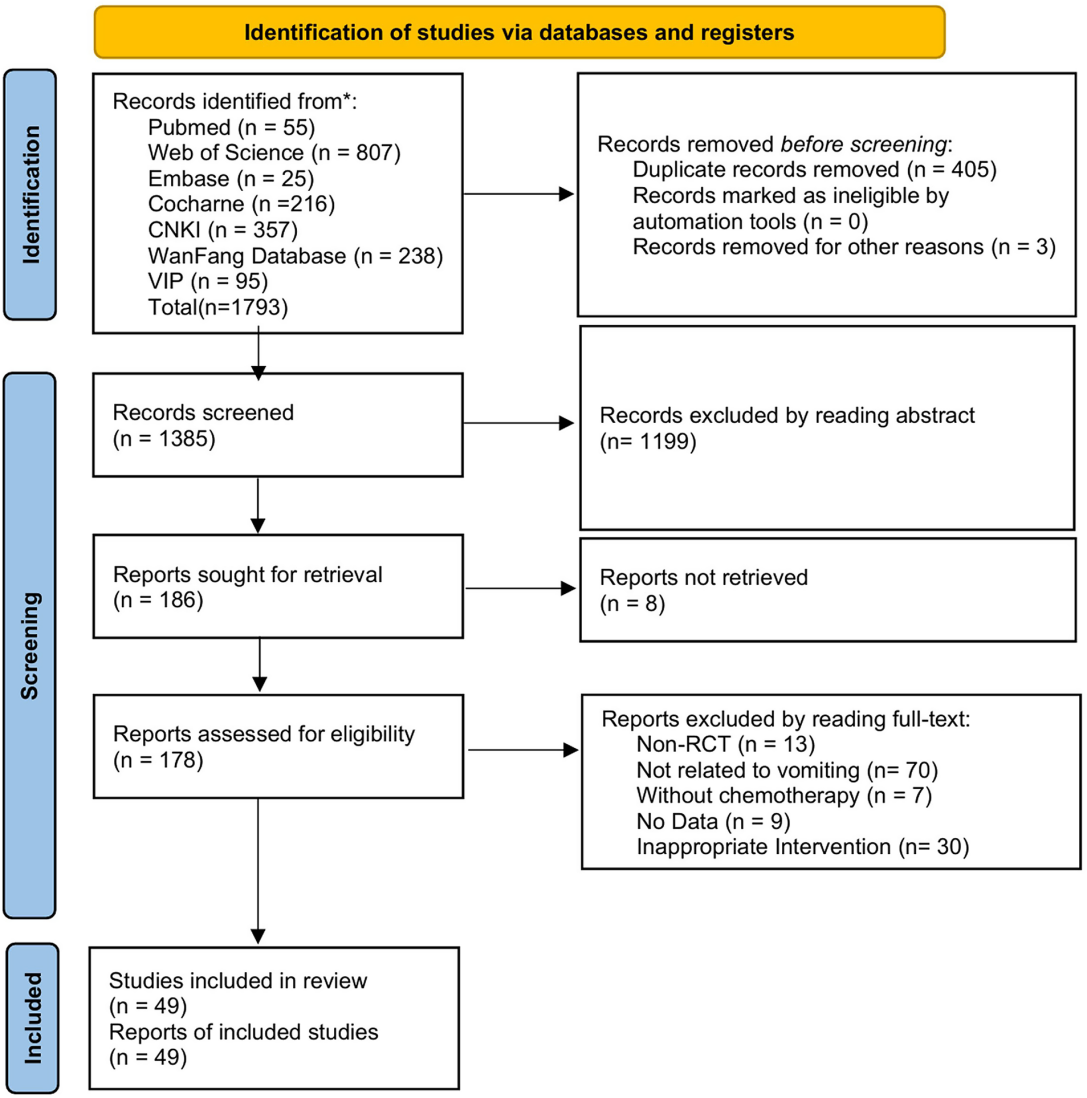
Flow diagram for data collection and analysis.

This study searched the following seven databases: PubMed, Cochrane Library, Web of Science, EMBASE, China National Knowledge Infrastructure (CNKI), Wanfang Data, and VIP Chinese Science and Technology Periodicals Database. The literature search covered the period from the establishment of each database to 30 September 2025, with no language restrictions. The final search was performed on 1 October 2025. During the search process, two types of search terms were adopted: Medical Subject Headings (MeSH) and free-text keywords. These two could be used independently or in combination to optimize search effectiveness. The detailed retrieval strategy was presented in Figure 1. Meanwhile, information on ongoing trials with unpublished data was obtained from clinical trial registration platforms such as the Chinese Clinical Trial Registry (ChiCTR) and Clinical Trials.gov was searched to obtain information on ongoing trials with unpublished data. For all potential literature, including relevant systematic reviews, researchers manually searched and sorted the reference lists to further identify additional relevant trials. If the literature contained incomplete data, the corresponding authors of the articles were contacted to provide the required additional information. The details of the search strategies for PubMed database are described in Table 1.

**Table 1:** Retrieval strategy in PubMed.

No.	Search items
#1	Neoplasms [Mesh]
#2	Tumors [Title/Abstract] OR Neoplasia [Title/Abstract] OR Neoplasias [Title/Abstract] OR Neoplasm [Title/Abstract] OR Tumor [Title/Abstract] OR Cancer [Title/Abstract] OR Cancers [Title/Abstract] OR Malignant Neoplasm [Title/Abstract] OR Malignancy [Title/Abstract] OR Malignancies [Title/Abstract] OR Malignant Neoplasms [Title/Abstract] OR Neoplasm, Malignant [Title/Abstract] OR Neoplasms, Malignant [Title/Abstract]
#3	#1 OR #2
#4	Acupuncture [Mesh] OR Acupuncture Therapy [Mesh]
#5	Acupuncture Treatment [Title/Abstract] OR Acupuncture Treatments [Title/Abstract] OR Treatment, Acupuncture [Title/Abstract] OR Therapy, Acupuncture [Title/Abstract] OR Acupotomy [Title/Abstract] OR Acupotomies [Title/Abstract] OR Electroacupuncture [Title/Abstract] OR electro acupuncture [Title/Abstract] OR acupuncture [Title/Abstract] OR Acupuncture Points [Title/Abstract] OR Acupuncture Point [Title/Abstract] OR Point, Acupuncture [Title/Abstract] OR Points, Acupuncture [Title/Abstract] OR Acupoints [Title/Abstract] OR Acupoint [Title/Abstract] OR P6 [Title/Abstract] OR P-6 [Title/Abstract] OR Meridians [Title/Abstract] OR Jing Luo [Title/Abstract] OR Luo, Jing [Title/Abstract] OR Jingluo [Title/Abstract] OR Ching Lo [Title/Abstract]
#6	#4 OR #5
#7	Drug Therapy [Mesh]
#8	Chemotherapy [Title/Abstract] OR Chemotherapies [Title/Abstract] OR Pharmacotherapy [Title/Abstract] OR Pharmacotherapies [Title/Abstract] OR Therapy, Drug [Title/Abstract] OR Drug Therapies [Title/Abstract] OR therapies, Drug [Title/Abstract]
#9	#7 OR #8
#10	Vomiting [Mesh]
#11	Emesis [Title/Abstract] OR nausea [Title/Abstract] OR retching [Title/Abstract]
#12	#10 OR #11
#13	Randomized controlled trial [Filter]
#14	#3 AND #6 AND #9 AND #12 AND #13

### 2.2 Inclusion criteria for literature

This study included literature meeting the following criteria: study design as RCT; participants as cancer patients at various stages of treatment; intervention as acupuncture therapy (including body acupuncture, auricular acupuncture, electroacupuncture, etc.); primary outcomes as the severity of nausea and vomiting assessed by validated tools. In the experimental group, the intervention might be acupuncture alone or acupuncture combined with conventional medications, while the control group received conventional medication treatment, or conventional medications combined with sham acupuncture. Therapies such as moxibustion, acupressure, acupoint injection, and transcutaneous electrical nerve stimulation (TENS) were excluded.

### 2.3 Outcome measures

This study’s primary outcome measures of this study included the incidence, severity, and frequency of nausea and vomiting, as well as validated scale scores such as the Rhodes Index of Nausea, Vomiting, and Retching (RINVR), the European Organization for Research and Treatment of Cancer Quality of Life Questionnaire (EORTC-QLQ-C30), and the Karnofsky Performance Status (KPS) scale.

### 2.4 Literature screening, data extraction, and bias analysis

After importing all the retrieved literature into EndNote software, two researchers (Chen Jiongli, Xu Fangyue) performed a duplicate removal exercise independently of each other. Then they conducted a preliminary screening of the remaining literature’s titles and abstracts. For literature deemed potentially eligible for inclusion after the preliminary screening, the full texts were further obtained and carefully reviewed. In the event of disagreements during the screening process, a third researcher (Li Rongrong) made the final decision. The overall process of literature retrieval and screening was shown in Figure 1.

Data extraction for this study was carried out independently by two researchers. The extracted content included publication information (first author, year of participant, country of publication), study design (sample size, age of participants, gender of participants, cancer type, grouping, control conditions, blinding), acupuncture protocol (acupoints used, number of treatments, treatment duration and frequency, intervention methods such as electroacupuncture, fire acupuncture and conventional acupuncture, etc.), and outcome measures. All of the above information was recorded in a standardized data extraction form. If the data reported in the literature was insufficient, the research team would take the initiative to contact the authors of the articles to obtain Supplementary material.

Two researchers independently assessed the risk of bias of the included literature using the Cochrane Risk of Bias (ROB) tool (20). This assessment covered random sequence generation, allocation concealment, blinding of participants and personnel, blinding of outcome assessors, completeness of outcome data, selective reporting of results, and other potential sources of bias. Based on the results of this assessment, the literature was classified into one of three categories: ‘low risk of bias’, ‘high risk of bias’, or ‘unclear risk of bias’.

### 2.5 Data analysis and result synthesis

We used R Project for statistical analysis and graph plotting. Based on observations from clinical practice, we adopted a random-effects model for all comparisons, taking into account potential clinical and methodological heterogeneity among different acupuncture treatment approaches. For continuous data, the statistical methods of mean difference (MD) statistical method was used. The risk ratio (RR) was applied to the analysis of dichotomous data. Meanwhile, the 95% confidence intervals (CI) were calculated for both the treatment and control groups. When trials included two types of acupuncture and control methods (e.g., acupuncture versus electroacupuncture versus no treatment), the data from the two acupuncture groups were split into two separate studies for analysis.

Heterogeneity was assessed using the *I*^2^ statistic. If the *I*^2^ value was less than 50%, a fixed-effects model was employed for data synthesis; if the *I*^2^ value exceeded 50%, this indicated significant heterogeneity. Potential causes were explored from both clinical and methodological perspectives, and explanations were provided or subgroup analyses and sensitivity analyses were conducted. When more than 10 studies were included in the meta-analysis, funnel plots were used to detect publication bias. If fewer than 10 studies were included, all studies were reviewed, and the results synthesized narratively.

### 2.6 Certainty of evidence

GRADE (Grading of Recommendations Assessment, Development and Evaluation) system was used to evaluate the reliability of the evidence. In accordance with the GRADE guidelines (21), the quality of the evidence was initially graded as ‘high’, but was progressively downgraded based on factors such as risk of bias, inconsistency, indirectness, and imprecision. Evidence grading was completed jointly by two researchers (Chen Jiongli, Zhang Lingyu). The GRADE assessment indicated that the overall quality of the results in this study was low. All outcomes were classified as moderate or low quality: the incidence of vomiting and nausea was rated as moderate certainty of evidence; the severity of vomiting and nausea were rated as low certainty of evidence; and the frequency of vomiting and nausea was rated as very low certainty.

## 3 Result

### 3.1 Characteristics of the study results

1,793 potentially relevant studies were retrieved. After removing duplicates (*n* = 405), 1,199 articles were excluded by reviewing titles and abstracts, followed by full-text screening of the remaining 186 articles. Ultimately, 49 RCTs (17, 18, 22–68) met the inclusion criteria. The general characteristics of the included studies is shown in Table 2. The results of the GRADE assessment is shown in Table 3. Most studies (92%, *n* = 45) were conducted in China, while the remainder were completed in the United States (*n* = 1) (67), Germany (*n* = 2) (64, 65), and Australia (*n* = 1) (68). Patient age ranged from 20 to 80 years, with sample size range from 12 to 127. Nine different acupuncture modalities were used to treat CINV in this study, including conventional acupuncture, electroacupuncture, wrist-ankle acupuncture, umbilical acupuncture, auricular acupuncture, fire acupuncture, intradermal acupuncture, and warm needle acupuncture. 30 studies used manual acupuncture with filiform needles, 10 studies combined electroacupuncture (17, 27–31, 44, 49, 57, 68), and one study combined acupuncture with thermotherapy (22). This covers a variety of acupuncture modalities and treatment protocols. Seven studies used sham acupuncture as the control group (17, 18, 27, 41, 46, 65, 68). Sixty-six distinct acupoints were utilized for CINV treatment, focusing on points along the Pericardium Meridian of Hand-Jueyin, Stomach Meridian of Foot-Yangming, and Conception Vessel. The three most frequently used acupoints were ST36, PC6, and CV12. Treatment was mostly administered once daily; session duration varied, with 30 min being the most common; and the treatment course ranged from 1 day to 20 weeks, with 14 studies lasting 3 days and 12 studies lasting 5 days.

**Table 2:** Characteristics of included systematic reviews on acupuncture for CINV in cancer patients.

Study ID	Sample size	Type of cancer	Intervention	Course of treatment	Acupoints	Instruments used
1	TG:62 CG:58	Breast cancer, malignant ovarian tumors, cervical cancer, endometrial cancer, lung cancer	TG: Acupuncture + Conventional therapy CG: Sham acupuncture + COnventional therapy	30 min, once a day	ST36, RN12, PC6, ST25, RN6, LR3	ECOG-PS; effective rate
2	TG:30 CG:30	Not report	TG: Warm acupuncture + conventional therapy CG: Conventional therapy	30 min, once a day, totally 6 days	LI4, ST36, PC6	Effective rate
3	TG:70 CG:70	Breast cancer	TG: Abdominal acupuncture + conventional therapy CG: Conventional therapy	30 min, twice a day, totally 3 days	RN12, RN10, RN6, RN4, ST25, SP15, ST24	Effective rate
4	TG:49 CG:47	Lung cancer	TG: Acupuncture + conventional therapy CG: Conventional therapy	30 min, once a day. During the retention period, the needle is inserted every 10 min	ST36, RN12, PC6	KPS; effective rate
	TG:44 CG:47	Lung cancer	TG: Acupuncture + conventional therapy CG: Conventional therapy	30 min,once a day. During the retention period, the needle is inserted every 10 min	ST36, RN12, PC6	KPS; effective rate
5	TG:27 CG:25	Tumors of the respiratory, digestive, urinary, reproductive and musculoskeletal systems	TG: Auricular acupuncture + conventional therapy CG: Conventional therapy	30 min, three times a day	CO6, CO2, CO4	Effective rate
6	TG:27 CG:25	Lung cancer, gastric cancer, colon cancer, rectal cancer, endometrial cancer, breast cancer, pancreatic cancer, osteosarcoma, kidney cancer, bile duct cancer, esophageal cancer	TG: Auricular acupuncture + conventional therapy CG: Conventional therapy	30 min, twice a day	The point of the vagus nerve of the ear, the point above the vagus nerve of the ear	Effective rate
7	TG:38 CG:34	Lung cancer, gastric cancer, colon cancer, rectal cancer, endometrial cancer, breast cancer, pancreatic cancer, osteosarcoma, kidney cancer, bile duct cancer, esophageal cancer	TG: Electroacupuncture + conventional therapy CG: Sham electroacupuncture+ Conventional therapy	1 h, twice a day	PC6, PC5	Frequency and degree of nausea and vomiting; Effective rate
8	TG:30 CG:30	Not report	TG: Electroacupuncture CG: Conventional therapy	30 min, once a day	ST36, RN12, PC6	R-INVR
9	TG:40 CG:40	Breast cancer	TG: Electroacupuncture + conventional therapy CG: Conventional therapy	20 min, once a day	LI4, PC6, ST36	Degree of vomiting and nausea
10	TG:30 CG:30	Lung cancer, stomach cancer, cholangiocarcinoma, ovarian cancer, rectal cancer, colon cancer, maxillary sinus cancer, non-Hodgkin’s lymphoma, breast cancer, cervical cancer, esophageal cancer, pancreatic cancer, cardia cancer	TG: Electroacupuncture CG: Conventional therapy	20 min, once a day	ST36, PC6, RN12, SP6	Effective rate
11	TG:127 CG:119	Not report	TG: Electroacupuncture + conventional therapy CG: Conventional therapy	30 min, once a day	ST36	Effective rate
12	TG:33 CG:33	Lung cancer, liver cancer, nasopharyngeal cancer	TG: Acupuncture + conventional therapy CG: Conventional therapy	30 min, once a day	EX-HN1, DU24, EX-HN3, HT7, SP6, ST36, RN12, PC6	KPS; WHO-QLQ-C30; degree of vomiting and nausea
13	TG:30 CG:30	Not report	TG: Acupuncture CG: Conventional therapy	50 min, twice a day	SP4, PC6, RN12, BL20, BL21	Effective rate
14	TG:20 CG:20	Not report	TG: Acupuncture+ Conventional therapy CG: Conventional therapy	30 min, twice a day	PC6, ST36, LI4, LV3, ST40	Effective rate; KPS
15	TG:33 CG:36	Breast cancer	TG: Fire needle+ Conventional therapy CG: Conventional therapy	Each acupoint should be pricked 7 times each time, for a total of 3 times	ST36, CV12, LU5, PC6, SP4, SP1, ST45	Effective rate; MAT
16	TG:28 CG:28	Breast cancer	TG: Intradermal needling + conventional therapy CG: Conventional therapy	The frequency of needle lifting replacement is once every 24 h. The acupuncture points on both sides are alternately buried. Press the acupoints for 30 min before the start of chemotherapy drug infusion, 30 min after the end of infusion, and for 30 s every hour	LI4, ST36, P6	Effective rate; FLIE; duration of vomiting and nausea
17	TG:30 CG:30	Intestinal cancer	TG: Intradermal needling + conventional therapy CG: Conventional therapy	The frequency of needle replacement is once every 48 h. 30 min before the start of chemotherapy drug infusion and 30 min after the end of infusion, press the acupoints for 2 min	LI4, PC6	Effective rate
18	TG:31 CG:30	Breast cancer	TG: Acupuncture CG: Conventional therapy	30 min, once a week	PC6, ST36, PC6, ST21, LV14, RN17, ST25, RN7, RN4, LV3, BL2, DU20, DU23, LI11, EX-HN3, LI4, SI11, DU14, BL18, DU4, BL20, DU1	Effective rate; KPS; WHO-QLQ-30
19	TG:24 CG:24	Breast cancer	TG: Intradermal needling + conventional therapy CG: Conventional therapy	The frequency of changing the lifting needle is once every 48 h, with intermittent kneading	LI4, ST36, PC6, LV3	Effective rate; duration of vomiting and nausea
20	TG:30 CG:30	Not report	TG: Wrist-ankle acupuncture + conventional therapy CG: Conventional therapy	30 min, once a day	Wrist ankle acupuncture upper 1st zone\upper 2nd zone (PC 6)	Effective rate; FLIE
21	TG:55 CG:56	Not report	TG: Wrist-ankle acupuncture + conventional therapy CG: Sham wrist and ankle acupuncture+ Conventional therapy	Retained for 20 h	Wrist ankle acupuncture upper 1st zone	R-INVR; KPS
22	TG:30 CG:31	Not report	TG: Acupuncture+ Conventional therapy CG: Conventional therapy	30 min, once a day	ST36, RN12, PC6	R-INVR; FACT-G; Traditional Chinese Medical Syndrome Scoring table for gastrointestinal diseases
23	TG:81 CG:80	Gastric cancer	TG: Acupuncture + Conventional therapy CG: Conventional therapy	30 min, once a day	ST36, RN12, PC6, ST25, SP4, BL20, BL21	WHO-QLQ-30; Effective rate; Degree of vomiting and nausea
24	TG:44 CG:44	Liver cancer	TG: Electroacupuncture + conventional therapy CG: Conventional therapy	20 min before the treatment	KI1	Effective rate; ECOG-PS
25	TG:29 CG:30	Lung cancer	TG: Acupuncture+ Conventional therapy CG: Conventional therapy	30 min, once a day	ST36, RN12, PC6, ST25	Effective rate; KPS; Degree of vomiting and nausea
26	TG:27 CG:30	Lung cancer, breast cancer, gynecological malignancies	TG: Acupuncture + conventional therapy CG: Sham acupuncture + conventional therapy	30 min, twice on the first day and once a day thereafter	ST36, RN12, PC6, ST25, LV13, RN6	ECOG-PS; traditional Chinese medicine symptom scoring scale
27	TG:32 CG:32	Breast cancer	TG: Acupuncture + conventional therapy CG: Conventional therapy	30 min, once a day	ST36	KPS; effective rate
28	TG:31 CG:12	Lung cancer, nasopharyngeal cancer, colorectal cancer, breast cancer, bone and flesh rumen cancer, liver cancer	TG: Acupuncture CG: Conventional therapy	30 min, 1–2 times a day	ST36, RN12, PC6	Degree of vomiting and nausea; effective rate
29	TG:48 CG:30	Breast cancer, stomach cancer, esophageal cancer, lung cancer, endometrial cancer, colorectal cancer, pancreatic cancer, ovarian cancer	T: Electroacupuncture C: Conventional therapy	30 min, once a day	ST36, RN12, PC6	Degree of vomiting and nausea; effective rate
30	TG:33 CG:33	Lung cancer, ovarian cancer, rectal cancer, gastric cancer, esophageal cancer, gallbladder cancer, thymic cancer, colon cancer, pancreatic cancer, breast cancer, bone cancer, hypopharyngeal cancer	TG: Acupuncture + conventional therapy CG: Conventional therapy	30 min, once a day	ST36, PC6, RN21, RN17	KPS; traditional Chinese medicine symptom scoring scale; effective rate
31	TG:43 CG:43	Lung cancer, breast cancer	TG: Acupuncture + conventional therapy CG: Conventional therapy	30 min. Twice on the first day and once a day thereafter	ST36, RN12, PC6, ST25, RN6, LV13	Traditional Chinese medicine symptom scoring scale; effective rate
32	TG:25 CG:25	Lung cancer, breast cancer, esophageal cancer, stomach cancer, intestinal cancer, ovarian cancer	TG: Acupuncture + conventional therapy CG: Conventional therapy	30 min, once a day	ST36, PC6, SP4, SP3, HT7	KPS; effective rate
33	TG:46 CG:48	Gastric cancer	TG: Acupuncture + conventional therapy CG: Conventional therapy	30 min, once a day	ST36, RN12, PC6, ST25, SP6, SP4	KPS; effective rate
34	TG:35 CG:35	Not report	TG: Acupuncture + conventional therapy CG: Conventional therapy	20–30 min, once a day	ST36, ST41, ST43, ST44, ST45	FLIE; effective rate
35	TG:38 CG:48	Lung cancer, ovarian cancer, breast cancer, gastric cancer	TG: Acupuncture + conventional therapy CG: Conventional therapy	30 min, three times a day	ST36, RN12, PC6	Degree of vomiting and nausea; effective rate; duration of vomiting
36	TG:21 CG:21	Nasopharynx cancer	TG: Acupuncture + conventional therapy CG: Conventional therapy	30 min, three times a week	ST21, ST25, ST36, RN10, RN12, RN13, RN17, BL2, LI4, PC6, SP4, Tu Shui point, Kai PI point, umbilical needle (Gen and Dui needles)	Degree of vomiting and nausea; effective rate; KPS
37	TG:50 CG:50	Lung cancer	TG: Electroacupuncture + conventional therapy CG: Conventional therapy	30 min, once a day	ST36	Effective rate
38	TG:38 CG:42	Lung cancer	TG: Acupuncture + conventional therapy CG: Conventional therapy	30 min, once a day	ST36	Effective rate
39	TG:25 CG:25	Lung cancer, breast cancer, gynecological malignancies	TG: Acupuncture + conventional therapy CG: Conventional therapy	30 min. Twice on the first day and once a day thereafter	ST36, RN12, PC6, SP4, ST40	Effective rate
40	TG:30 CG:30	Ovarian cancer	TG: Acupuncture + conventional therapy CG: Conventional	30 min, once a day	ST36, ST21, RN12, KI21	KPS; MAT; traditional Chinese medicine symptom scoring scale
41	TG:44 CG:43	Lung cancer, ovarian cancer, breast cancer, bladder cancer, mediastinal neuroendocrine cancer	TG: Acupuncture + conventional therapy CG: Conventional	30 min, once a day	ST36, RN12, PC6	Effective rate
42	TG:107 CG:105	Not report	TG: Wrist-ankle acupuncture CG: Conventional therapy	2 h, once a day	The upper 1st zone on both sides, the lower left 1st zone, and the lower left 2nd zone	FLIE; effective rate
43	TG:28 CG:28	Lung cancer	TG: Acupuncture + Conventional therapy C: Conventional therapy	2 h, once a day	ST36	Effective rate
44	TG:23 CG:23	Ewing’s sarcoma, rhabdomyosarcoma, osteosarcoma, undifferentiated sarcoma, synovial sarcoma	TG: Acupuncture + conventional therapy CG: Conventional therapy	Each time for 20–45 min	ST36, RN12, PC6, LI4	Dosage of antiemetic drugs; episodes of nausea and vomiting
45	TG:41 CG:39	Multiple myeloma, breast cancer, non-Hodgkin’s lymphoma, lipomatosis, Hodgkin’s lymphoma	TG: Acupuncture + conventional therapy CG: Placebo acupuncture + conventional therapy	20 min, once a day	PC6	Effective rate
46	TG:28 CG:28	Gastric cancer	TG: Acupuncture + conventional therapy CG: Conventional therapy	30 min, once a day	ST36, ST25, PC6, SP6, GB21	WHO-QOL-100; episodes of vomiting
47	TG:119 CG:116	Breast cancer	TG: Electroacupuncture + conventional therapy CG: Sham electroacupuncture + conventional therapy	15 or 30 min, once a day	ST36, PC6, LI4	Effective rate
48	TG:37 CG:33	Breast cancer	TG: Acupuncture + conventional therapy CG: Conventional therapy	20 min, once a day	ST36	Episodes of vomiting
49	TG:14 CG:16	Breast cancer	TG: Electroacupuncture + conventional therapy CG: Sham electroacupuncture + Conventional therapy	20 min, 2 days before each cycle, once a day	ST36, PC6, LI4	MAT; EFFECTIVE rate

TG, Treatment Group; CG, Control Group; KPS, Karnofsky Performance Status; ECOG, Eastern Cooperative Oncology Group Performance Status; MAT, Multinational Association of Supportive Care in Cancer Antiemesis Tool; WHOQOL, World Health Organization Quality of Life Instrument; FLIE, Functional Living Index – Emesis; RINVR, Rhodes Index of Nausea, Vomiting, and Retching; FACT-G, Functional Assessment of Cancer Therapy – General.

**Table 3:** GRADE summary of findings table.

No of studies	Study design	Risk of bias	Inconsistency	Indirectness	Imprecision	Other considerations	Acupuncture alone or acupuncture combined with standard medication	Standard medication alone or standard medication combined with sham acupuncture	Relative (95%CI)	Absolute (95%CI)	Certainty
Vomiting percentage											
47	Randomized trials	Serious^a^	Not serious	Not serious	Not serious	None	361/1,952 (18.5%)	590/1,896 (31.1%)	**RR 0.57** (0.49–0.67)	**134 fewer per 1,000** (from 159 fewer to 103 fewer)	⨁⨁⨁◯ Moderate^a^
Frequency of vomiting											
4	Randomized trials	Serious^b^	Very serious^b^	Not serious	Not serious	None	118	114	–	MD **3.71 lower** (6.29 lower to 1.13 lower)	⨁◯◯◯ Very low^b^
Vomiting severity											
8	Randomized trials	Serious^b^	Very serious^b^	Not serious	Not serious	None	435	404	–	MD **0.92 lower** (1.57 lower to 0.28 lower)	⨁◯◯◯ Very low^b^
Nausea percentage											
33	Randomized trials	Serious^c^	Not serious	Not serious	Not serious	None	310/1,381 (22.4%)	564/1,356 (41.6%)	**RR 0.55** (0.45–0.66)	**187 fewer per 1,000** (from 229 fewer to 141 fewer)	⨁⨁⨁◯ Moderate^c^
Frequency of nausea											
2	Randomized trials	Serious^b^	Very serious^b^	Not serious	Not serious	None	67	69	–	MD **2.5 lower** (5.91 lower to 0.91 higher)	⨁◯◯◯ Very low^b^
Nausea severity											
8	Randomized trials	Serious^b^	Serious^d^	Not serious	Not serious	None	315	307	–	MD **0.95 lower** (1.43 lower to 0.47 lower)	⨁⨁◯◯ Low^b,d^

CI, Confidence Interval; MD, Mean Difference; RR, Risk Ratio.

^a^Complete blinding cannot be achieved in acupuncture studies. Fourteen studies did not blind participants, operators, or outcome assessors, while the blinding status for participants, operators, and outcome assessors was unclear in most studies (*n* = 32). Additionally, allocation concealment status was unclear in some studies.

^b^Insufficient number of included studies.

^c^Complete blinding cannot be achieved in acupuncture studies. Eleven studies did not blind participants, operators, or outcome assessors, while the blinding status for participants, operators, and outcome assessors was unclear in most studies (*n* = 35). Additionally, allocation concealment status was unclear in some studies.

^d^High heterogeneity with a *p* < 0.001.

### 3.2 Risk of bias in studies

**Figure 2:**
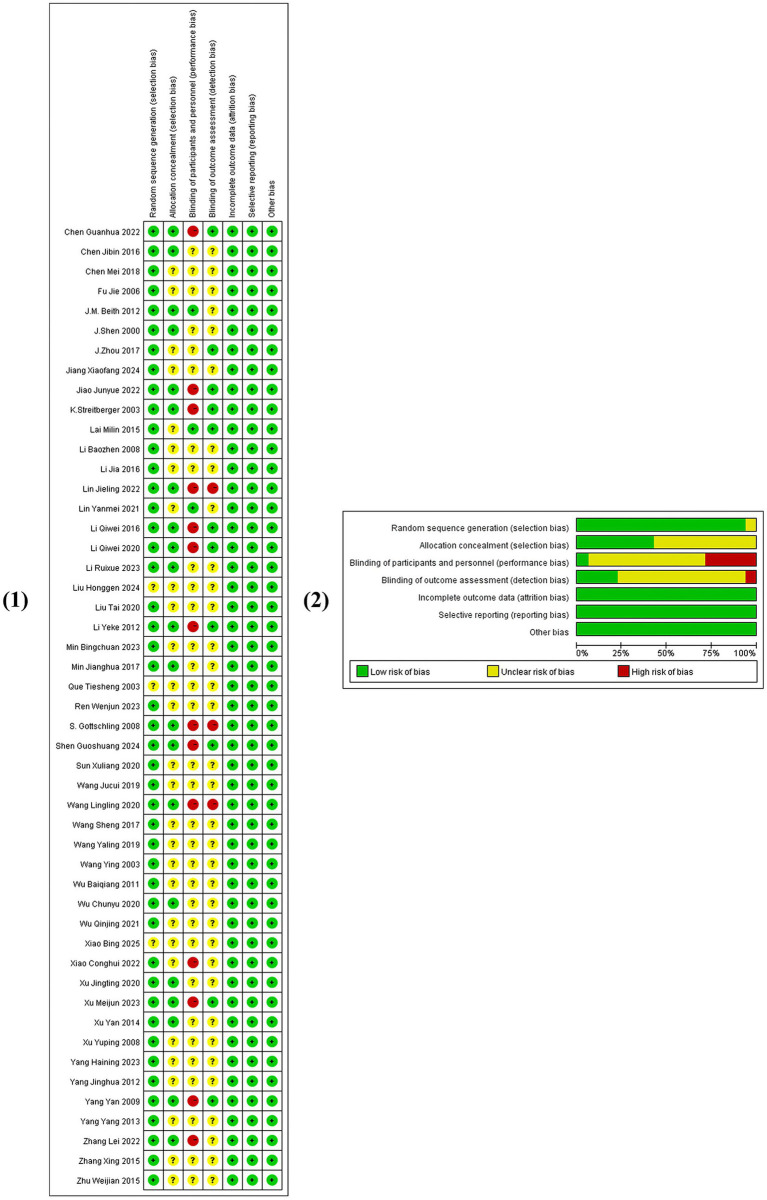
Risk of bias of the included systematic reviews by ROB tool.

Regarding selection bias, the random sequence generation process was adequate and appropriate in 46 studies (94%). The randomization process was unclear in the remaining three studies (6%) (48, 51, 53): these studies claimed to be “randomized” but did not report the method used for generating the random sequence. Allocation concealment was implemented in 21 studies (43%), while it was unclear in the remaining 28 studies (57%). Due to patients and acupuncturists being unable to be blinded during acupuncture treatment, no fully double-blind trials were identified among the included literature. Only 15 studies (31%) (17, 18, 23, 31, 35, 36, 38, 41, 42, 46, 47, 65–68) involved blinded third-party assessors or used sham acupuncture as a control group to blind patients; however these methods carried a high risk of unblinding. Four studies (8%) (32, 52, 56, 64) did not employ any blinding methods, and the blinding status was unclear in 30 studies (61%). Attrition bias was low in all studies, as participant loss to follow-up was controlled within a reasonable range. The conclusions of all the included studies were consistent with the reported outcome measures, with no selective reporting of results. No other potential sources of bias were identified in the other studies (as shown in Figure 2).

### 3.3 Therapeutic effect of acupuncture for CINV

The pooled statistics from the meta-analysis on acupuncture for the control of CINV are summarized below. Funnel plot analysis revealed the presence of publication bias among the included studies. The trim-and-fill method was applied to correct the results and fill in the missing data. Following correction, the effect size of acupuncture for CINV control increased, and the 95% CI remained to the left of the effect line, indicating statistical significance. This had no impact on the overall conclusions of the study. Additionally, significant heterogeneity was observed across some studies. Subgroup analysis and sensitivity analyses were performed to evaluate the robustness of this study’s results.

#### 3.3.1 Therapeutic effect of vomiting

**Figure 3:**
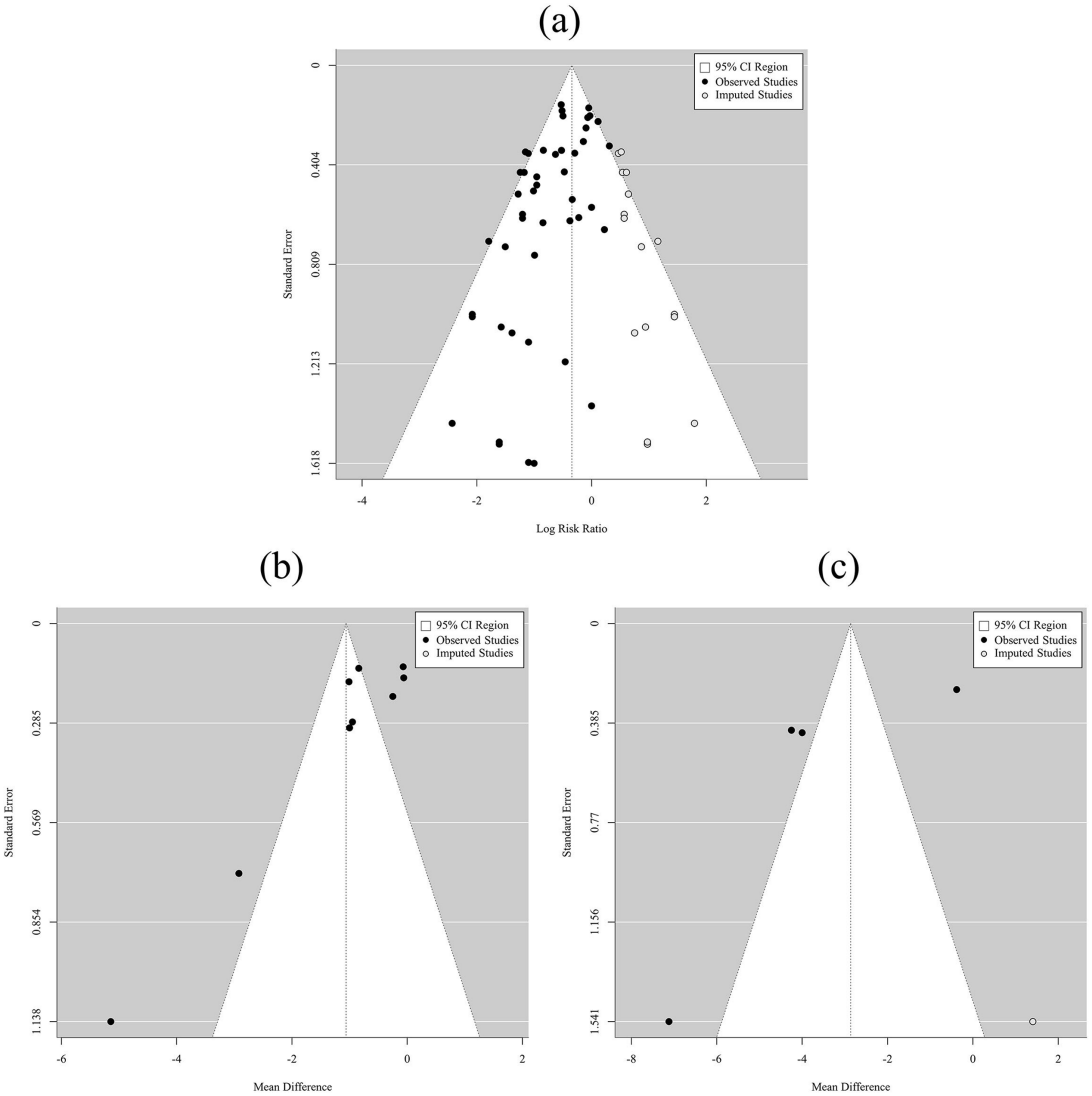
Funnel plot of the acupuncture-mediated vomiting control for **(a)** vomiting percentage, **(b)** vomiting severity, **(c)** frequency of vomiting.

**Figure 4:**
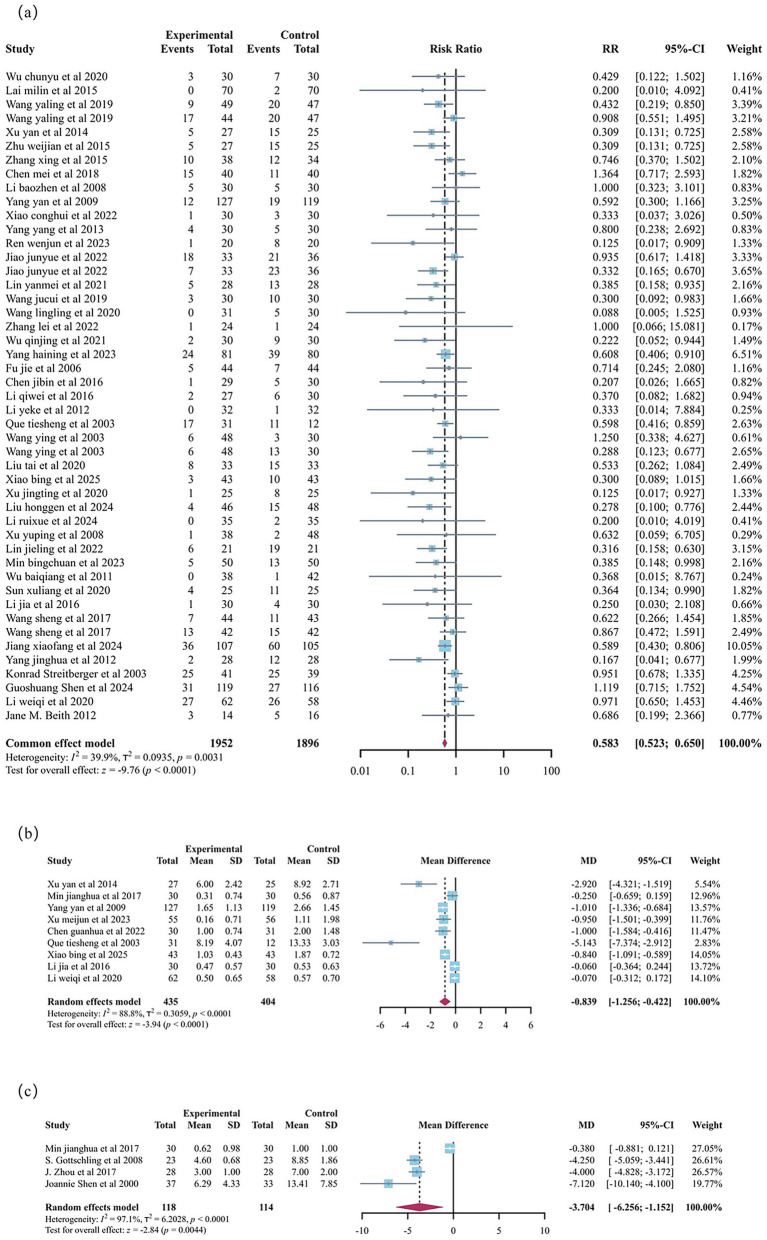
Forest plot of the acupuncture-mediated vomiting control for **(a)** vomiting percentage, **(b)** vomiting severity, **(c)** frequency of vomiting.

**Figure 5:**
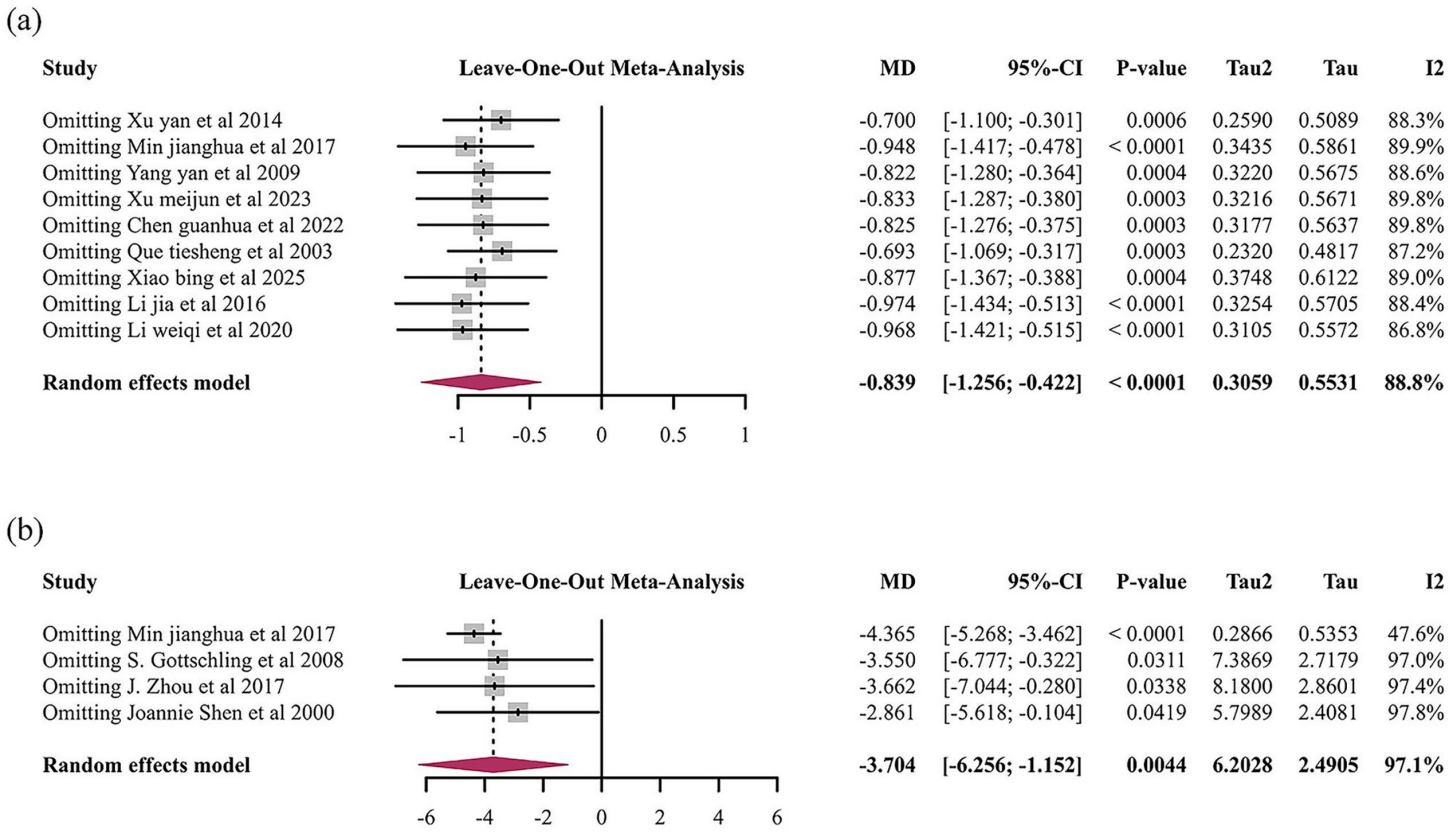
Sensitivity analysis of the acupuncture-mediated vomiting control for **(a)** vomiting severity, **(b)** frequency of vomiting.

The funnel plot of vomiting incidence indicated a certain degree of publication bias, which remained within an acceptable range (Figure 3a). By contrast, the number of studies reporting vomiting severity and vomiting frequency was small (*n* < 10), and the asymmetric distribution of data points suggested the presence of high publication bias and substantial heterogeneity among the included studies (Figures 3b,c). After treatment, the incidence of vomiting (RR = 0.583, 95% CI: 0.523–0.650, *p* = 0.0031, *I*^2^ = 39.9%, moderate certainty of evidence) (Figure 4a), vomiting severity score (MD = −0.839, 95% CI: −1.256 to −0.422, *p* < 0.0001, *I*^2^ = 88.8%, low certainty of evidence) (Figure 4b), and vomiting frequency(MD = −3.704, 95% CI: −6.256 to −1.152, *p* < 0.0001, *I*^2^ = 97.1%, very low certainty of evidence) (Figure 4c), all demonstrated that all MD values and 95% CIs fell on the same side of the null line. This indicated that acupuncture may control vomiting symptoms and has a therapeutic effect. However, significant heterogeneity was observed in the frequency and severity of vomiting. Due to the insufficient number of included studies, a subgroup analysis could not be performed; therefore, a sensitivity analysis was conducted, while the results revealed that excluding any single study did not result in substantially change in the overall pooled effect, as illustrated in Figure 5.

#### 3.3.2 Therapeutic effect of nausea

**Figure 6:**
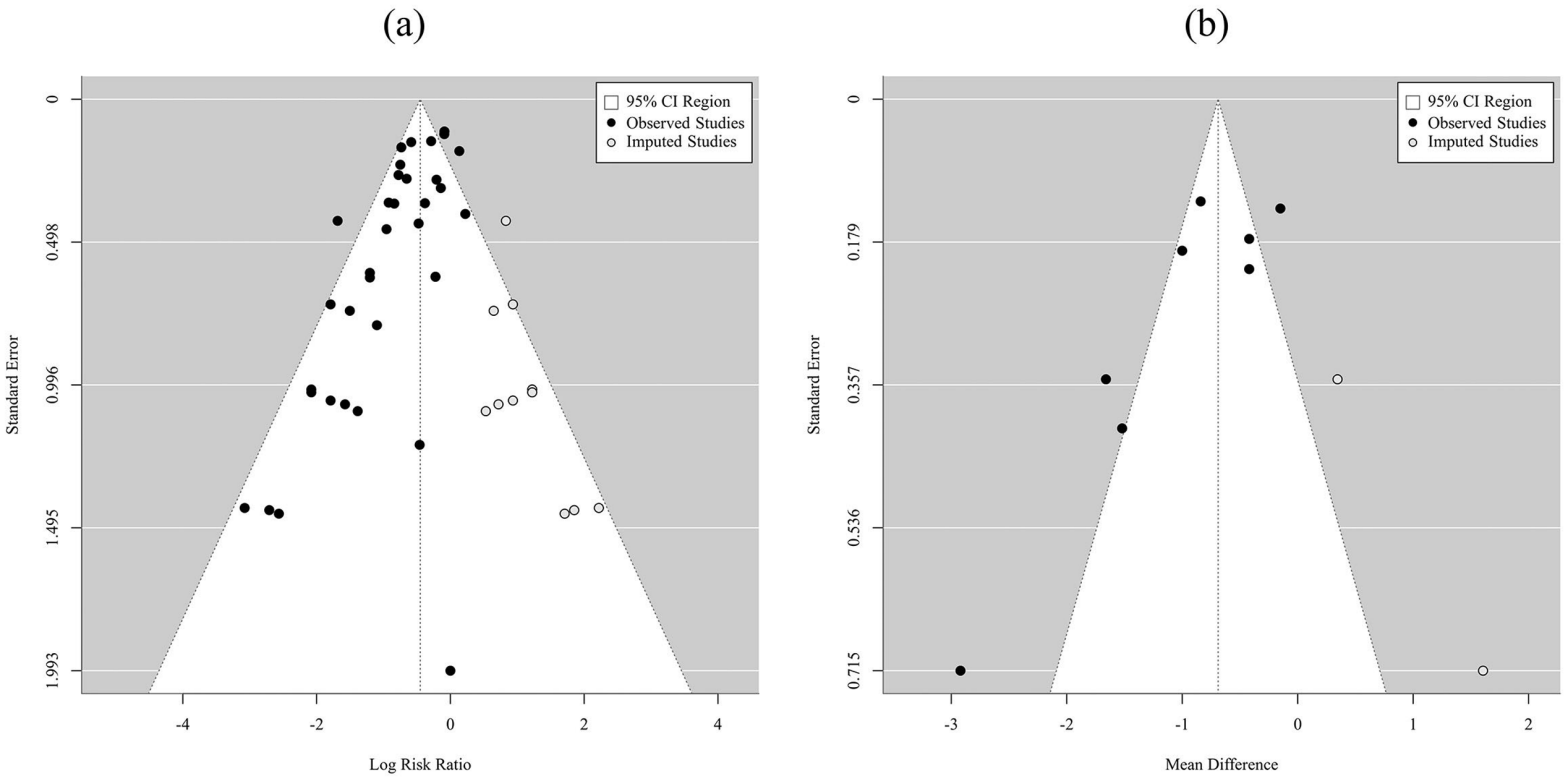
Funnel plot of the acupuncture-mediated nausea control for **(a)** nausea percentage, **(b)** nausea severity.

**Figure 7:**
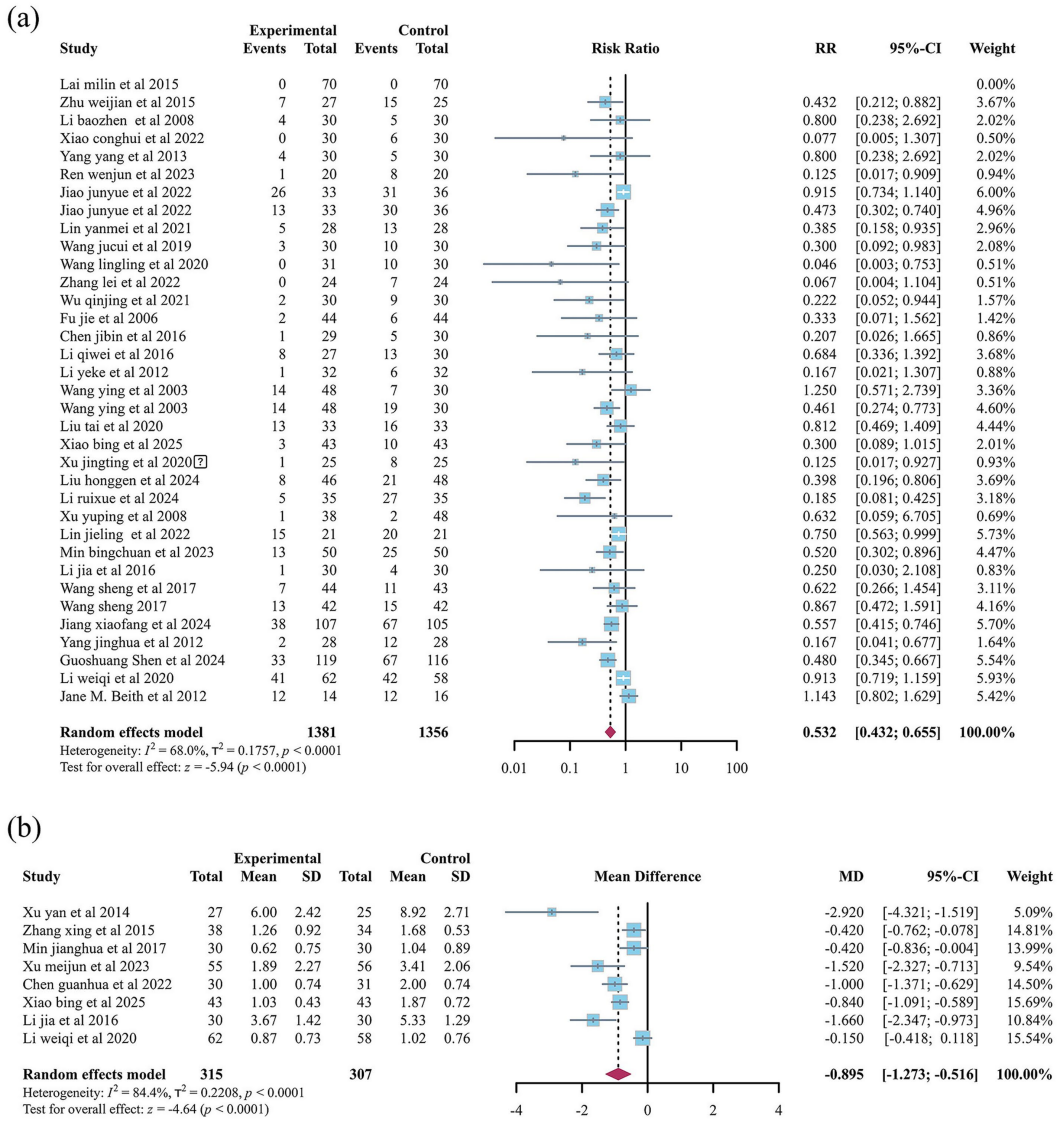
Forest plot of the acupuncture-mediated nausea control for **(a)** nausea percentage, **(b)** nausea severity.

**Figure 8:**
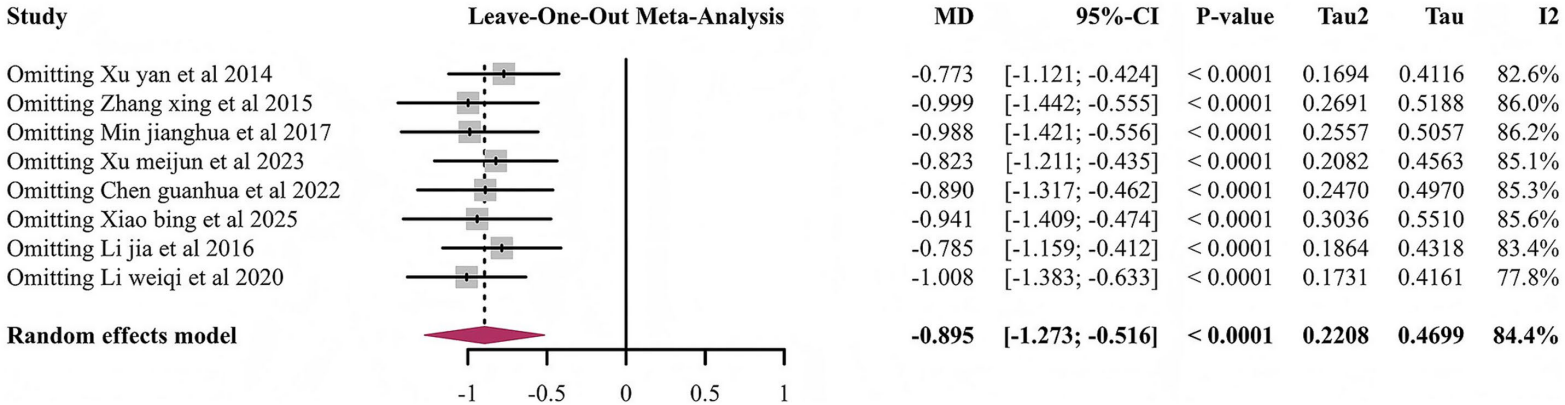
Sensitivity analysis of the acupuncture-mediated nausea control for nausea severity.

As shown in the funnel plot, there was a certain degree of publication bias in the incidence of nausea, while the heterogeneity among studies remained within an acceptable range (Figure 6a). Studies reporting nausea severity may be at a relatively high risk of publication bias or heterogeneity; therefore, the reliability of their results should be interpreted with caution (Figure 6b). Regarding nausea: the incidence of nausea (RR = 0.532, 95% CI: 0.432–0.655, *p* < 0.0001, *I*^2^ = 68.0%; moderate certainty of evidence) (Figure 7a) and severity score (MD = −0.895, 95% CI: −1.273 to −0.516, *p* < 0.0001, *I*^2^ = 84.4%; low certainty of evidence) (Figure 7b), these findings indicated that acupuncture intervention could control nausea. Additionally, subgroup analysis could not be conducted for nausea severity, as the number of included studies was fewer than 10. To determine the impact of each study on the results, a leave-one-out sensitivity analysis was conducted. The results demonstrated that the findings for nausea severity were robust, whereas the stability of the results for nausea frequency was poor (Figure 8).

#### 3.3.3 Subgroup analysis

**Figure 9:**
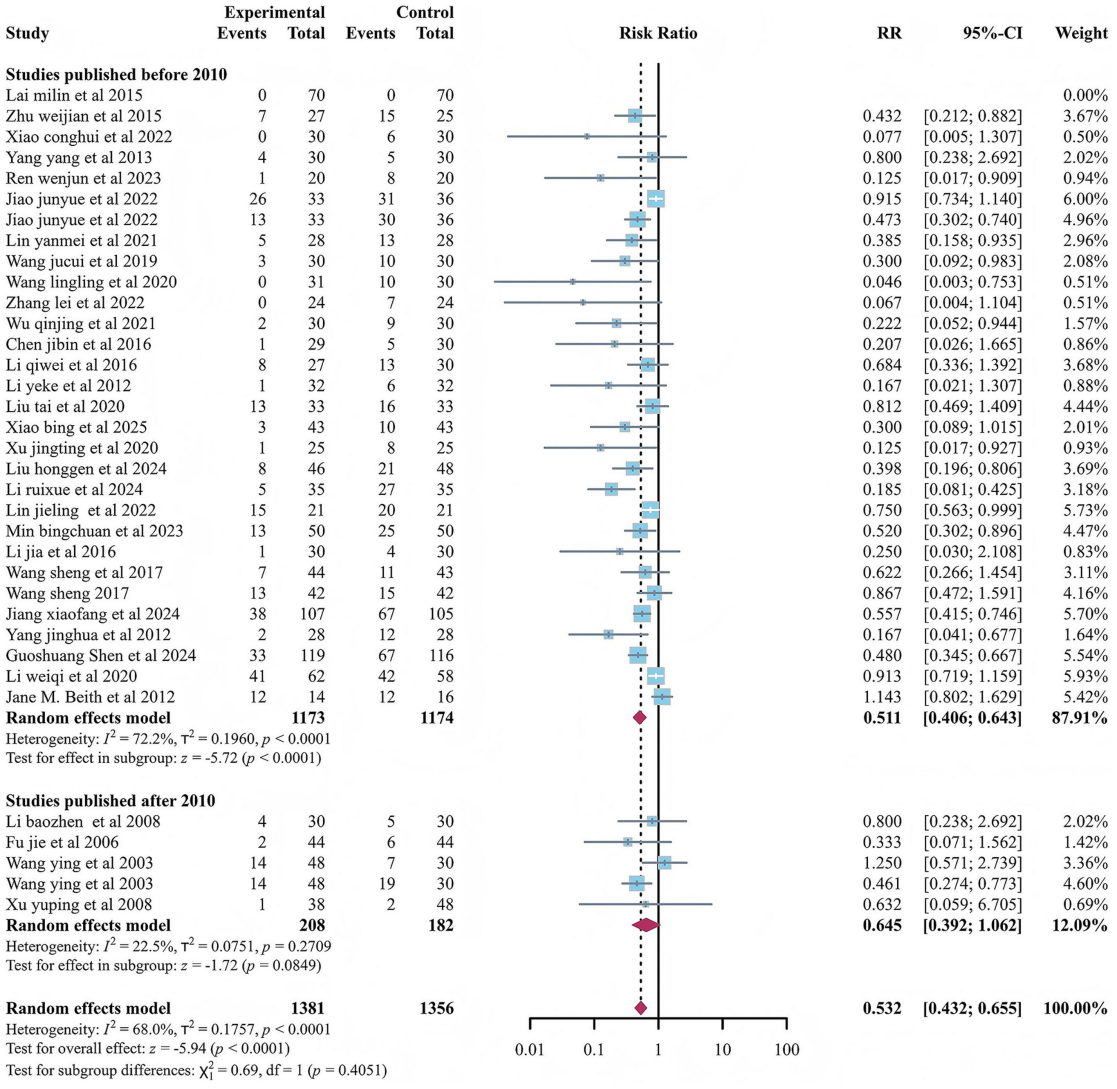
Subgroup analysis of the Acupuncture-Mediated Nausea Control for nausea percentage based on the year.

Given that *I*^2^ > 50% in the above results, a subgroup analysis was performed for the incidence of nausea. The results showed that the pooled effect of studies published before 2010 (RR = 0.511, 95% CI: 0.406–0.643, *p* < 0.0001, *I*^2^ = 72.2%) was significant with high heterogeneity, while the pooled effect of studies published after 2010 (RR = 0.645, 95% CI: 0.392–1.062, *p* = 0.2709, *I*^2^ = 22.5%) was non-significant with low heterogeneity (Figure 9); no statistically significant difference was observed between the subgroups, but still indicated that acupuncture intervention can reduce risk of nausea. Additional subgroup analyses were performed based on the severity and frequency of vomiting. In terms of vomiting severity, there was no statistically significant difference among subgroups (*p* = 0.4954), with high heterogeneity across all subgroups (Supplementary Figure 1). Regarding vomiting frequency, no significant inter-subgroup difference was observed (*p* = 0.8709), and low heterogeneity was detected in the acupuncture group (*I*^2^ = 0%) (Supplementary Figure 2). Subgroup analyses were also conducted for the incidence and severity of nausea. For nausea incidence, there were no statistically significant differences in therapeutic effects among different intervention subgroups (*p* = 0.432), with low heterogeneity in the acupuncture group (*I*^2^ = 61.2%) (Supplementary Figure 3). In terms of nausea severity, no significant difference was found between subgroups (*p* = 0.8599), and the acupuncture group presented low heterogeneity (*I*^2^ = 0%) (Supplementary Figure 4). Except for the subgroup analysis of nausea incidence, the total number of included studies in the other three subgroup analyses was less than 10. Therefore, the clinical validity of the corresponding results should be interpreted with caution.

## 4 Discussion

This systematic review and meta-analysis demonstrates that acupuncture as an adjunctive therapy improves the complete response rate for CINV compared with conventional treatment alone, although it cannot completely replace pharmacotherapy.

Based on the RCTs currently, this review provides a relatively comprehensive body of evidence for the efficacy of acupuncture in preventing CINV. A total of 49 studies involving 4,133 participants were included. While previous systematic reviews have evaluated the effectiveness of non-penetrative modalities such as moxibustion, acupressure and TENS for CINV (69–71), the present study is the first to restrict its scope strictly to acupuncture therapy itself. Our findings suggest that acupuncture can reduce the incidence, frequency and severity of nausea and vomiting following chemotherapy. However, biases exist in blinding procedures, allocation concealment and outcome assessment, which compromise the reliability of the results. Compared with a prior systematic review and meta-analysis focusing on acupuncture (72), our findings are consistent with those review’s conclusions regarding antiemetic efficacy, with both demonstrating beneficial effects. Nevertheless, a discrepancy emerges regarding the alleviation of nausea: our study confirms that acupuncture can also alleviate post-chemotherapy nausea, which differs from the outcomes of the aforementioned research. This discrepancy may stem from the intrinsic differences between nausea and vomiting. Nausea is a subjective sensation, influenced by individual and psychological factors, making it difficult to measure objectively (73). In contrast, vomiting is an objective event, and its occurrence can easily be determined. The efficacy of interventions targeting vomiting can therefore be more readily assessed (74). For this reason, clinical studies often measure vomiting more frequently than nausea (75), and patients tend to confuse nausea with other types of gastric discomfort when reporting symptoms. Furthermore, inadequate blinding designs (e.g., unblinding caused by interactions between patients and acupuncturists) can particularly interfere with the assessment of subjective outcomes such as nausea. This may be a key factor contributing to inconsistent results across studies. In conclusion, future studies must adopt validated disease-specific scales for the targeted nausea assessment (76, 77) and the rigorously implement of blinding protocols are crucial for future studies to yield more reliable conclusions.

The results of the remaining four studies showed high heterogeneity, warranting subgroup analysis, except for the incidence of vomiting. However, subgroup analysis could only be performed for the incidence of nausea (*n* = 32). As there were fewer than 10 studies included for the three outcomes (i.e., frequency of vomiting, severity of vomiting, and severity of nausea), subgroup analysis was not feasible. Therefore, sensitivity analysis was conducted for these four outcomes to verify the robustness of the results.

This study identified ST36, PC6, and CV12 as the predominant acupoints in CINV management, accounting for the majority of selections among 66 documented points. This consistent clinical preference is supported by both data mining evidence (78) and well-characterized physiological mechanisms. ST36 primarily exerted its effects through peripheral modulation of gastrointestinal motility via muscarinic (*M*2/*M*3) and adrenergic (*β*1/*β*2) receptor activation (79–83). Additionally, PC6 demonstrated comparable efficacy to pharmacological antiemetics (84, 85) through its unique ability to modulate key neurotransmitters-simultaneously elevating *β*-endorphin levels in cerebrospinal fluid while reducing peripheral 5-HT concentrations (86, 87). CV12 regulated the level of serum 5-HT and relieved gastrointestinal smooth muscle spasm, serving as a pivotal acupoint for the treatment of various digestive disorders (88). The above acupoints can stimulate the activities of the sympathetic and parasympathetic nerves, regulates the secretion of neurotransmitters (5-HT, *β*-endorphin), regulates gastrointestinal motility and nerve conduction, and regulates the brain-gut connection, thereby alleviating and exerting the antiemetic effect (89–91).

The present study has three distinct strengths. First, our analysis focused exclusively on acupuncture therapy without incorporating other adjunctive treatments, which minimized the potential confounding effects and thus provided more reliable clinical evidence for the efficacy of acupuncture in CINV management. Second, this study systematically summarized the selection principles of therapeutic acupoints and meridians, which is conducive to developing more optimized clinical protocols for CINV. In addition to synthesizing the current evidence regarding the effectiveness of acupuncture for CINV, this systematic review employed the GRADE approach to assess the quality of evidence. Furthermore, sensitivity analysis was performed to determine whether the results varied with predefined explanatory variables.

Nevertheless, several limitations should be acknowledged. First, at the data level, some included studies failed to distinguish between acute and delayed CINV—the latter is generally more severe and refractory to control (92). To ensure consistency, we extracted data at the end of treatment, based on the principle that the intervention group demonstrated a relatively high control rate. This practice may have introduced bias in estimating acupuncture’s efficacy. This practice might have led to biases in the estimation of acupuncture efficacy. Second, in the evidence grading process, the quality of evidence was downgraded due to identified high risks of bias, imprecision or inconsistency in results, which may have collectively contributed to a conservative estimation of the actual effect of acupuncture; Methodologically, as a complex intervention, acupuncture poses inherent challenges to the implementation of adequate blinding of patients and practitioners in RCTs (93). Methodological limitations were noted regarding blinding procedures in the included studies, with only 7% (*n* = 49) implementing adequate participant blinding. While sham acupuncture represents the current standard control in acupuncture trials, emerging evidence suggests it may exert physiological effects that complicate the interpretation of placebo responses (94). This is particularly relevant for subjective outcomes such as nausea severity scores, where unblinded assessment may introduce measurement bias. Moreover, the baseline characteristics of the included studies revealed substantial heterogeneity in the scales employed to assess subjective nausea and vomiting outcomes. Importantly, no consensus has been established regarding the optimal selection of outcome measures. Such heterogeneity in measurement approaches reduces both the comparability of treatment effects across studies and the clinical interpretability of pooled results (95, 96). Addationally, the subgroup analyses indicated that low heterogeneity of conventional acupuncture in key outcome indicators, including vomiting frequency, severity of nausea, and incidence of nausea. These findings indicated that despite technical discrepancies in manual acupuncture protocols, such interventions could yield more consistent antiemetic effects, which might be attributed to standardized acupoint selection (e.g., PC6, ST36) and comparable acupuncture manipulation across included trials. In contrast, high heterogeneity was observed for electroacupuncture across all outcomes, which was likely caused by uncontrollable confounding variables such as stimulation parameters (frequency and intensity), electrode placement and treatment duration.

Moreover, due to the poor quality of the included literature, the risk of inadequate allocation concealment and outcome assessment blinding was unclear. Regarding publication bias, the asymmetric funnel plots suggested that negative results might have been unpublished (Figures 3, 6). However, the Egger’s test could not be performed due to the small number of included studies (*n* < 10). Additionally, the sample sizes for some outcome indicators, especially frequency and severity of vomiting and nausea, were too small, for subsequent subgroup analyses to be conducted following sensitivity analysis, resulting in imprecise effect estimates. Finally, while this study exclusively included randomized controlled trials, its must be noted that 92% of these were conducted in China. This geographic concentration raises important considerations regarding cultural context and patient expectations. Specifically, the higher cultural acceptance and familiarity with acupuncture in traditional Chinese medicine-practicing regions may systematically influence treatment outcomes through mechanisms such as enhanced placebo effects and practitioner-patient interactions. These factors may potentially affect both the magnitude and generalizability of our findings, particularly when applied to populations with different cultural backgrounds and healthcare systems. Hence, the generalizability of these findings to non-Chinese populations should be interpreted with caution due to potential cultural and expectancy-related confounders. Future international multicenter, high-quality RCTs are warranted to validate the efficacy of acupuncture across diverse populations.

Additionally, the NCCN categorizes chemotherapeutic agents into four emetogenic risk levels (high, moderate, low, minimal). While acupuncture may show significant efficacy in high-emetogenic-risk cases where conventional antiemetics often fail, its benefits could be limited in moderate- or low-risk settings (14, 96). Chemotherapy regimens also contribute to baseline heterogeneity among patients, such as physical impairment, myelosuppression, and anemia, potentially increasing acupuncture-related safety risks (89). Thus, rigorously designed trials are needed to evaluate acupuncture’s safety and efficacy across varying chemotherapy protocols.

## 5 Conclusion

This study confirms that acupuncture is effective as an adjunctive therapy for CINV. While future large-scale, rigorously designed RCTs are warranted to further validate these findings, the current evidence provides a robust rationale for its integration into clinical practice.

## Data Availability

The original contributions presented in the study are included in the article/Supplementary material, further inquiries can be directed to the corresponding author.

## Footnotes

1
https://www.crd.york.ac.uk/PROSPERO/view/CRD420251102130
