# Supplementary material for: Efficacy and safety of acupuncture for chemotherapy-induced nausea and vomiting: a systematic review and meta-analysis of randomized controlled trial
Source: Front Neurol. 2026 Jul 3;17:1774507. doi: 10.3389/fneur.2026.1774507 (PMC13375487; doi:10.3389/fneur.2026.1774507)
Supplement: Supplementary file 1 [file Table_1.docx]

**
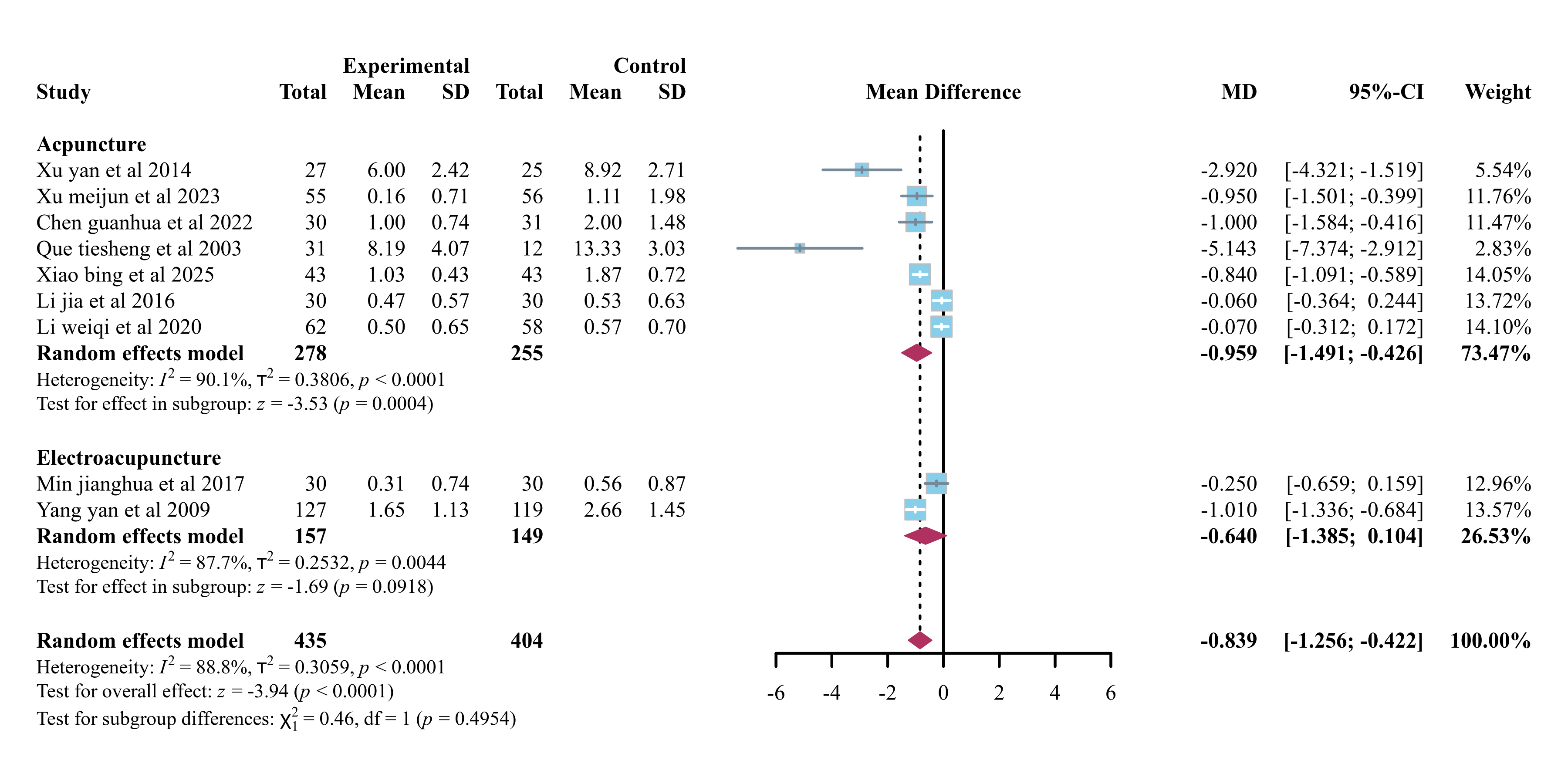
**

**eFigure 1 Subgroup analysis of different acupuncture methods on vomiting severity**


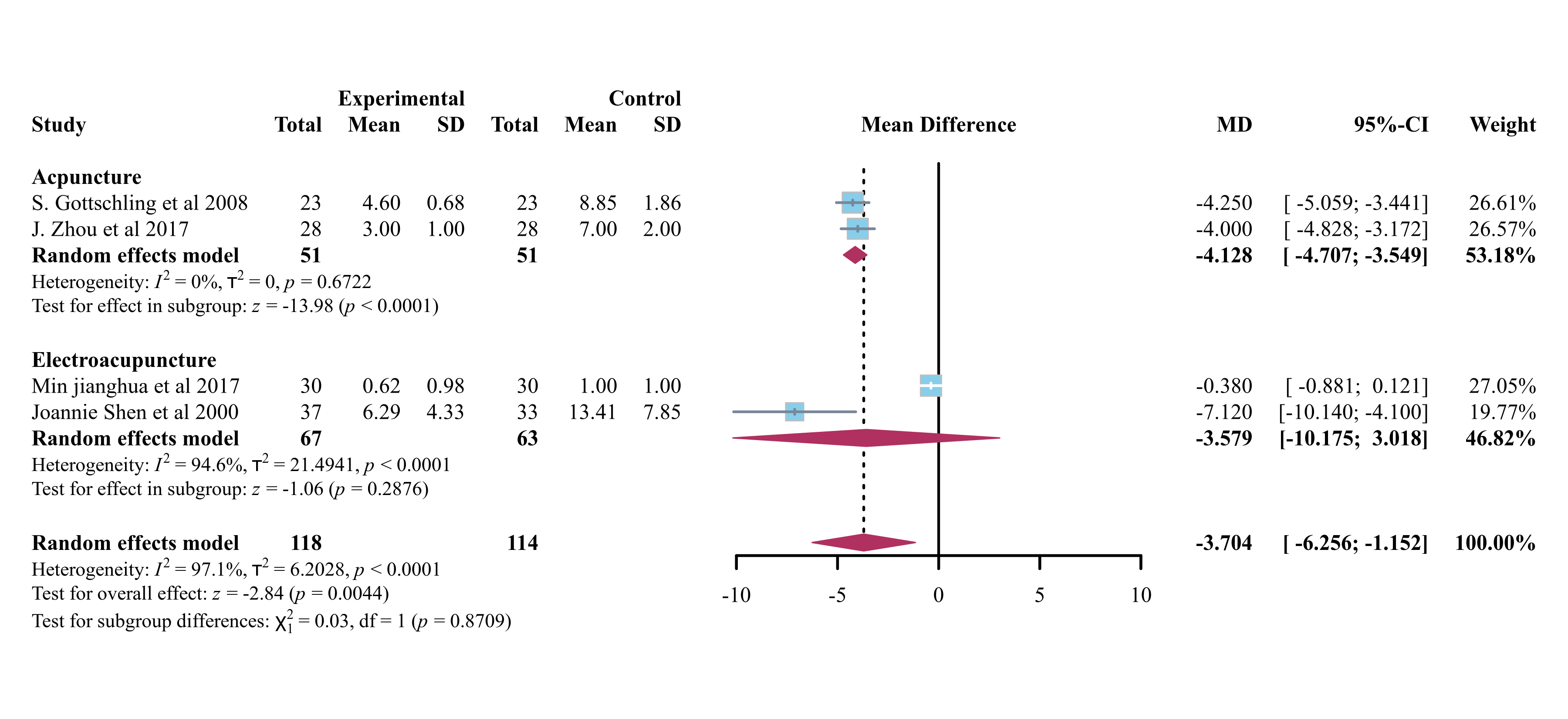


**eFigure 2** **Subgroup analysis of different acupuncture methods on frequency of vomiting**


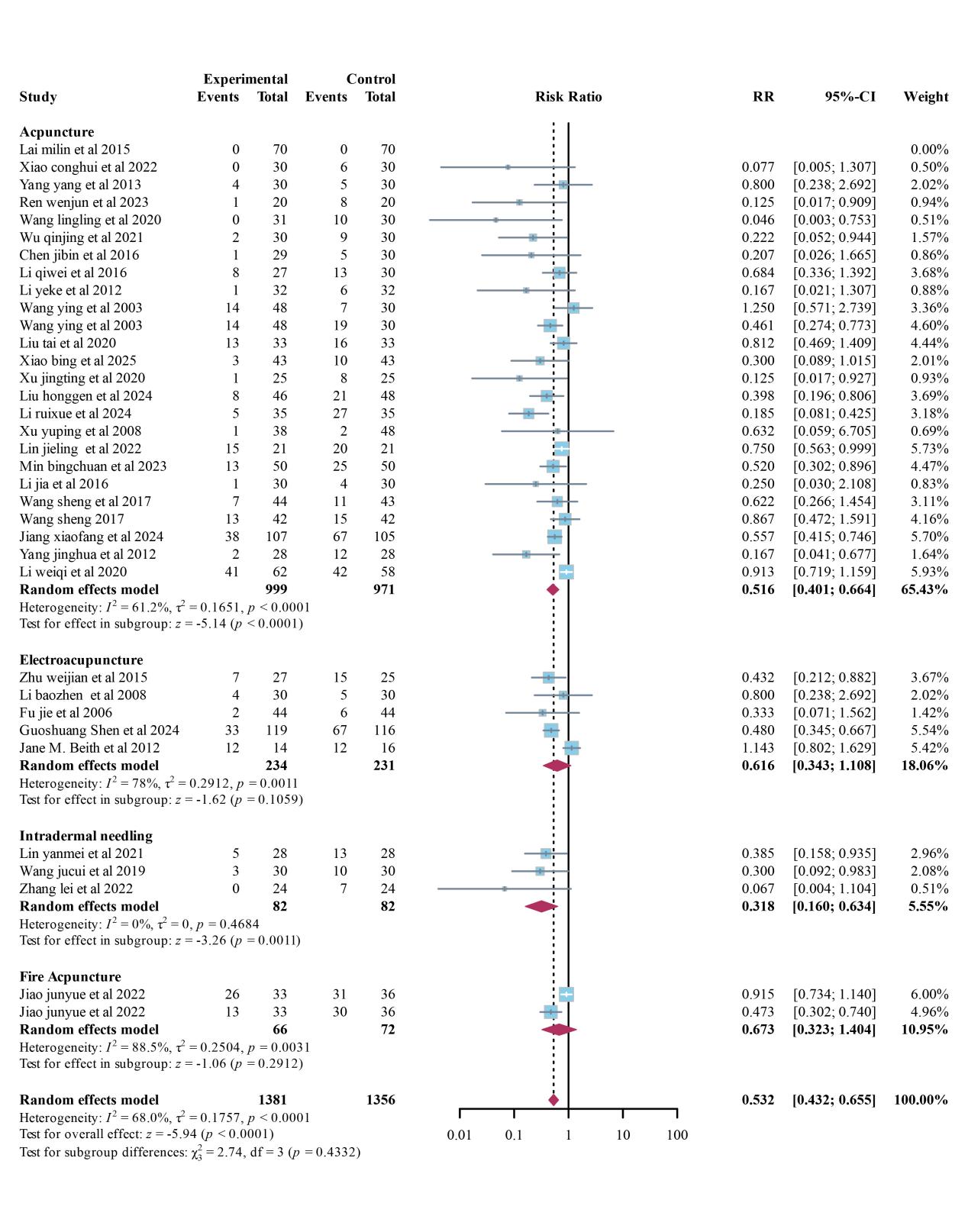


**eFigure 3 Subgroup analysis of different acupuncture methods on incidence of nausea**


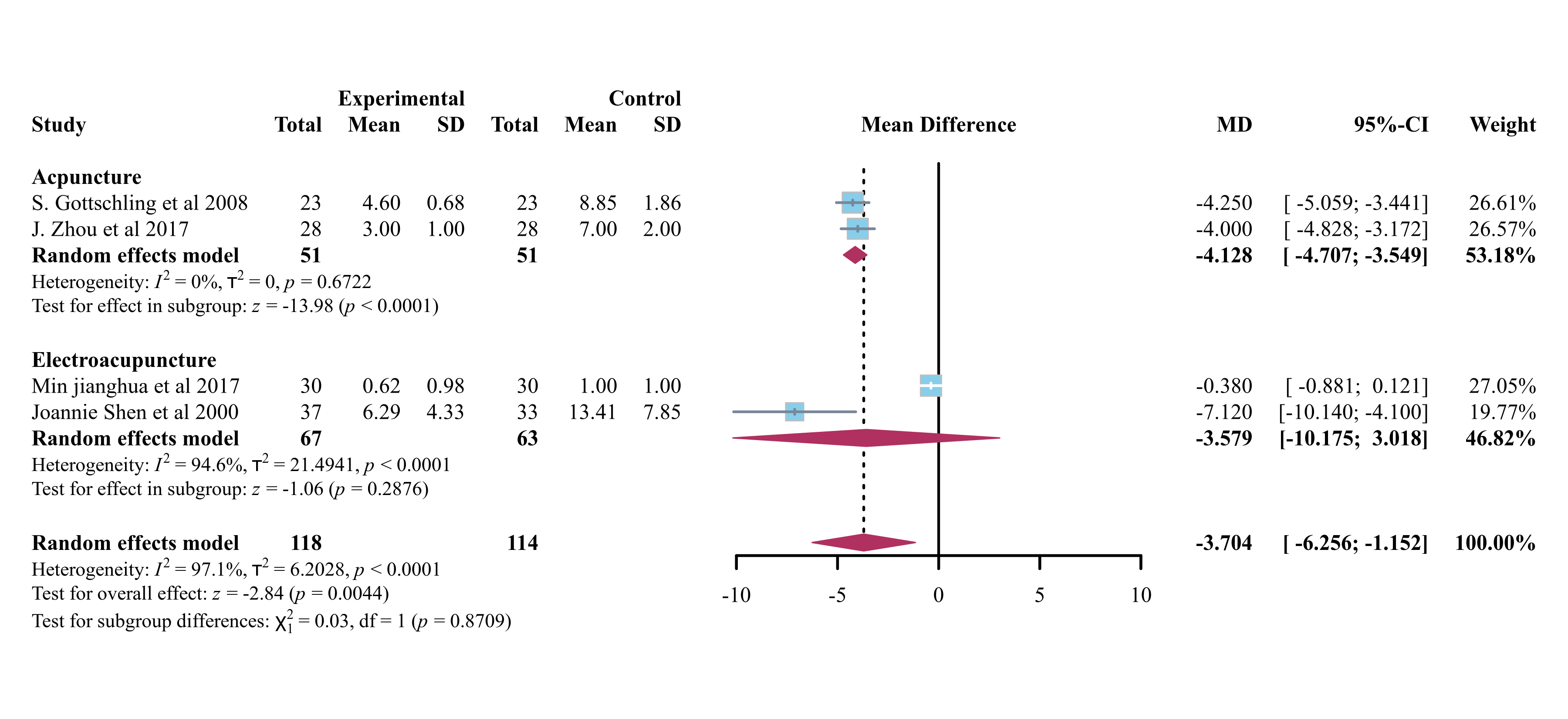


**eFigure 4 Subgroup analysis of different acupuncture methods for nausea severity**
